# Bicaval obstruction complicating right atrial tuberculoma:the diagnostic value of Cardiovascular MR

**DOI:** 10.1186/1532-429X-10-60

**Published:** 2008-12-20

**Authors:** Ibrahim Al-Nasser, Ashraf M Anwar, Youssef FM Nosir, Mohammed AR Chamsi-Pasha, Aref Ajam, Aymen Alqiriaqri, Hassan Chamsi-Pasha

**Affiliations:** 1 Department of Radiology*, King Fahd Armed Forces Hospital, Jeddah, Saudi Arabia; 2 Department of Cardiology, King Fahd Armed Forces Hospital, Jeddah, Saudi Arabia; 3 Department of Medicine, King Fahd Armed Forces Hospital, Jeddah, Saudi Arabia; 4 Department of medicine, King Abdul Aziz University, Jeddah, Saudi Arabia

## Abstract

Cardiac tuberculosis is rare and usually involves the pericardium. Myocardial tuberculoma is a very rare occurrence and only a few cases were reported.

We describe the use of cardiovascular magnetic resonance in the diagnosis of a rare case of cardiac tuberculoma involving the right atrium which was complicated by a bicaval obstruction. The patient made a remarkable improvement with the anti-tuberculous treatment. To our knowledge, this complication has never been reported in relation to cardiac tuberculoma.

## Introduction

Tuberculosis can involve a multitude of organ tissues but generally affects the respiratory tract. Involvement of the atria with cardiac tuberculoma is exceptionally rare [1]. We report a case of a bicaval obstruction in a young patient with cardiac tuberculoma.

The case highlights not only the description of a previously unreported complication of cardiac tuberculoma, but also the use of Cardiovascular Magnetic Resonance (CMR) in establishing the diagnosis.

## Case presentation

A 25-years old male, previously well, presented with fatigue, sweating, shortness of breath on exertion and weight loss over a 4-months period. During the last two weeks prior to admission he developed atypical chest pain, cough and hemoptysis. There was no relevant past medical history.

Clinical examination was unremarkable apart from right apical bronchial breathing with no signs of superior vena cava (SVC) obstruction. ECG showed incomplete RBBB, sinus tachycardia with left anterior fascicular block. Chest X ray demonstrated right apical infiltration. Both complete blood count & biochemistry profile were normal. Erythrocyte sedimentation rate was 65 and C – reactive protein 58. Sputum culture was positive for acid fast bacilli. Bronchial biopsy showed granulomatous inflammation of bronchial mucosa in keeping with tuberculosis. Protein C & S were normal

**Figure 1:**
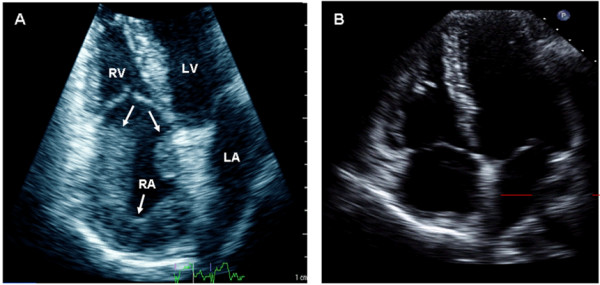
**Two-dimensional echocardiography showed the large right atrial (RA) mass (arrows) on apical 4-chambers view (A) and almost complete resolution after the treatment (B)**.

**Figure 2:**
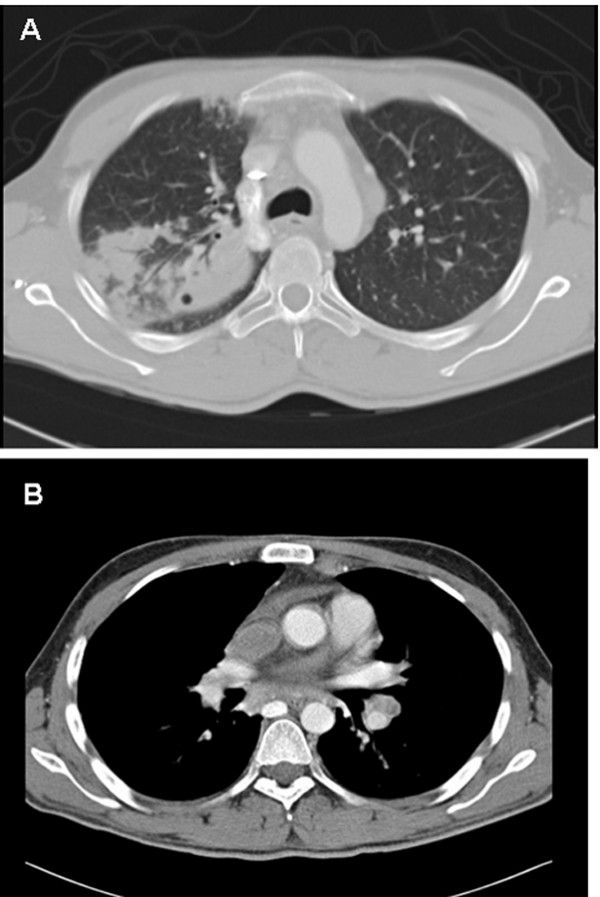
**Chest CT scan showed right lung consolidation (A), with enlarged mediastinal lymph nodes (B)**.

Transthoracic echocardiography showed dilated right atrium (RA) with a large "horse-shoe" mass involving most of the RA wall (Figure 1a). CT scan showed confluent ill-defined areas of consolidation with multiple thin-walled cavitations at the upper lobe of the right lung and multiple mediastinal and hilar lymph nodes (Figure 2).

**Figure 3:**
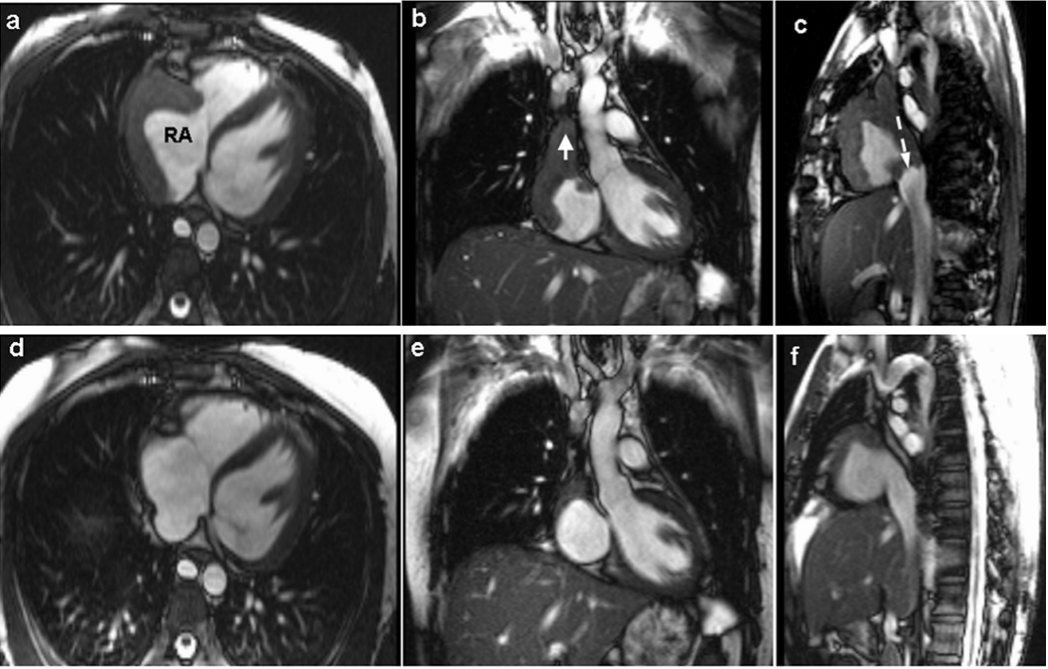
**CMR balanced gradient-echo images in axial (a), sagittal (b) and coronal views (c) showed the mass (M) in right atrium (RA), superior vena cava (short arrow), and inferior vena cava (long arrow) before treatment and the corresponding images 12-months after (d, e, f)**.

**Figure 4:**
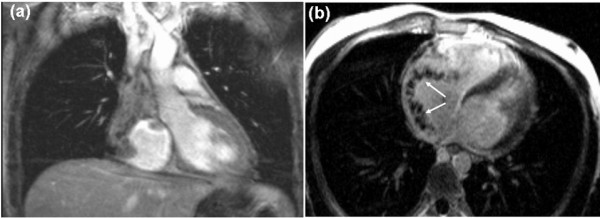
**CMR early (a) and late (b) gadolinium-enhanced images in coronal and axial sections showed non-homogenous enhancement within the mass (arrows)**.

CMR showed extensive soft tissue mass involving the right atrial wall circumferentially with complete SVC obstruction and partial inferior vena cava (IVC) obstruction. The azygos vein was dilated consistent with SVC obstruction (Figure 3a, b, c). The late gadolinium-enhanced images showed diffuse enhancement within the mass indicating necrosis or inflammation (Figure 4). Cardiac biopsy was requested but the patient declined.

A diagnosis of cardiac tuberculoma predominately involving the RA and both SVC and IVC with possible secondary thrombus was made. The patient received anti-tuberculosis as well as anticoagulation therapy and there was remarkable improvement of symptoms. Follow up echocardiography (Figure 1b) and CMR (Figure 3d, e, f) 12 months after the start of therapy showed significant reduction of the mass and partial resolution of the IVC obstruction but the SVC obstruction remains.

## Discussion

Single or multiple cardiac tuberculomas are rare and most often observed in the right heart chambers, particularly in the RA wall. They are usually well circumscribed and sharply demarcated from the surrounding parenchyma [1,2]. They may erode the underlying myocardium, resulting in ulcers that in turn cause thrombus formation and subsequent embolism [2].

To our knowledge, this is the first report of a bicaval obstruction secondary to cardiac tuberculoma. Most cases of superior vena cava obstruction are caused by malignant mediastinal neoplasms, especially bronchogenic carcinoma and less frequently by a non-malignant lesion such as mediastinal tuberculosis or goiter [3]. Walker et al reported a case of an acute bicaval obstruction as a result of intracapsular haemorrhage in a right atrial myxoma [4].

This case showed an unusual cardiac complication of tuberculosis not only involving the RA but also causing an obstruction of both superior and inferior vena cava. CMR played an important role in the initial diagnosis and the follow up of this patient.

## Consent

Written informed consent was obtained from the patient for publication of this case report and accompanying images. A copy of the written consent is available for review by the Editor-in-chief of this journal.

## Competing interests

The authors declare that they have no competing interests.

## Authors' contributions

IAN interpreted and analyzed MRI data. AMA drafted and formatted the manuscript according to journal instructions. AA was the primary physician of the patient. MAR was a medical intern collected the clinical data. YFN performed and reported the echocardiographic findings. AA contributed to the analysis of CT and MRI findings. HCP reviewed the whole manuscript before submission.
